# The COVID-19 Pandemic Economic Implications in Iran: A National Survey Assessing Catastrophic Health Expenditures

**DOI:** 10.1155/ipid/8854646

**Published:** 2025-07-23

**Authors:** Enayatollah Homaie Rad, Mohammad Hajizadeh, Shahrokh Yousefzadeh-Chabok, Fatemeh Keihanian, Vahid Yazdi-Feyzabadi, Leila Kouchakinejad-Eramsadati, Hedayat Salari, Atefeh Esfandiari, Hamed Zandian, Masoud Lotfizadeh, Hakimeh Mostafavi, Masoud Arefnezhad, Reza Esmaeili, Mandana Saki, Bakhtiar Piroozi, Sajad Delavari, Mahmood Karimy

**Affiliations:** ^1^Department of Health Economics, Social Determinants of Health Research Center, Trauma Institute, Guilan University of Medical Sciences, Rasht, Iran; ^2^Department of Health Economics, School of Health Administration, Dalhousie University, Halifax, Nova Scotia, Canada; ^3^Department of Neurosurgery, Guilan Road Trauma Research Center, Trauma Institute, Guilan University of Medical Sciences, Rasht, Iran; ^4^Department of Health Policy, Guilan Road Trauma Research Center, Trauma Institute, Guilan University of Medical Sciences, Rasht, Iran; ^5^Department of Health Policy, Health Services Management Research Center, Institute for Futures Studies in Health, Kerman University of Medical Sciences, Kerman, Iran; ^6^Department of Disaster Medicine, Guilan Road Trauma Research Center, Trauma Institute, Guilan University of Medical Sciences, Rasht, Iran; ^7^Department of Health Policy and Management, School of Medicine, Bushehr University of Medical Sciences, Bushehr, Iran; ^8^Department of Health Policy, Centre for Public Health and Wellbeing, School of Health and Social Wellbeing, College of Health, Science and Society, University of the West of England, Bristol, UK; ^9^Department of Health Policy, Social Determinants of Health Research Center, Ardebil University of Medical Sciences, Ardabil, Iran; ^10^Department of Social Health, Social Determinants of Health Research Center, Shahrekord University of Medical Sciences, Shahrekord, Iran; ^11^Department of Health Policy, Health Equity Research Center (HERC), Tehran University of Medical Sciences, Tehran, Iran; ^12^Department of Health Economics and Management, School of Public Health, Tehran University of Medical Sciences, Tehran, Iran; ^13^Department of Health Economics and Management, School of Public Health, Zabol University of Medical Sciences, Zabol, Iran; ^14^Department of Public Health, School of Health, Social Development and Health Promotion Research Center, Gonabad University of Medical Sciences, Gonabad, Iran; ^15^Department of Nursing, Social Determinants of Health Research Center, Lorestan University of Medical Sciences, Khorramabad, Iran; ^16^Department of Health Policy, Social Determinants of Health Research Center, Kurdistan University of Medical Sciences, Sanandaj, Iran; ^17^Department of Health Policy, Health and Human Resource Research Center, Shiraz University of Medical Sciences, Shiraz, Iran; ^18^Department of Health Education and Promotion, Social Determinants of Health Research Center, Saveh University of Medical Sciences, Saveh, Iran

**Keywords:** catastrophic costs, coping strategies, inpatient costs, Iran, outpatient costs, the COVID-19

## Abstract

**Introduction:** The COVID-19 pandemic caused many financial crises in households worldwide. This study aimed to quantify the COVID-19-related catastrophic costs (CCC) in Iran during the pandemic.

**Methods:** In this national survey, a total of 2006 households from 10 provinces of Iran were selected using a multistage random cluster sampling. The data were collected on COVID-19 prevention, inpatient, outpatient, and income loss costs, and the household income and wealth information using a validated researcher-constructed questionnaire in 2022. We calculated the probability of the CCC with and without coping strategies. We analyzed data using logistic regression models and estimated the CCC for other provinces using the 2021 Household Income and Expenditures Survey.

**Results:** The CCC was 3.19% with coping strategies and 5.38% without coping strategies. The CCC positively correlated with the COVID-19 inpatient (*β* = 2.324, 95% CI [1.65 to 2.997]) and outpatient (*β* = 1.797, 95% CI [1.165 to 2.430]) service utilization. Access to the basic (*β* = −0.687, 95% CI [−1.248 to −0.109]) and complementary (*β* = −1.201, 95% CI [−2.612 to 0.210]) health insurance decreased the risk of the CCC. The highest and lowest probabilities of estimated CCC were observed in Sistan and Baluchistan (8.57%) and Tehran (2.1%) provinces, respectively.

**Conclusion:** The COVID-19 pandemic imposed an additional financial burden on households. The pandemic provided important lessons for health policymakers about the effectiveness of the health financing protection system during the crisis and the scarcity of health resources. Supply and demand of services are unbalanced in the outbreaks, and insurance systems might fall into failure due to the shortage of services, black markets, and price inflation.

## 1. Introduction

The COVID-19 pandemic was one of the most critical issues for the world's health systems. The pandemic had unprecedented global impact since the World War II in both higher and lower income countries [1, 2]. The COVID-19 could affect the household economy in three ways of prevention costs such as masks, and herbal drugs for prevention of infection; diagnosis and treatment expenses, including outpatient visits, hospital stays, and medications; and income loss due to illness, quarantine, or caregiving duties [3, 4]. These combined factors heightened the risk of catastrophic health expenditures (CHE), especially in countries with limited health financing protections. All of these issues might lead to CHE. The CHE is an essential measure of the failure of health systems. Health systems must ensure the financial protection of individuals and households who experience financial hardship due to out-of-pocket health spending [5, 6]. The catastrophic expenditure estimates the burden of health expenditure on households. The rate of households' exposure to CHE shows the number of households that fall into the financial hardship due to the diseases [7]. CHE occurs when healthcare spending exceeds a portion of household income, often defined as more than 20% of total income. CHE is a famous indicator of financial protection, signaling when households are pushed into economic hardship due to health-related costs [7]. Achieving a near-zero percentage of households facing catastrophic is an important goal of health systems. To control CHE in the households the World Health Organization (WHO) suggests that the out-of-pocket payments must be less than 15% [8]. Due to the COVID-19-related lockdowns, many households had missed their work or decreased their time working, spent money on protective equipment, and purchased additional health and medical services [9]. These payments might increase the health-related out-of-pocket payments and CHE. Due to economic sanctions imposed during COVID-19, the financial impact of the pandemic may have been particularly severe in Iran, which was among the countries significantly affected by the health crisis [10]. Economic sanctions have an inflationary effect, particularly in countries heavily reliant on foreign trade revenues. During the COVID-19 pandemic, this dynamic became more severe. Supply-side shocks from the pandemic resulted in inflation, undermining the capacity of both households and governments to manage the crisis which might affect healthcare provision due to economic instability [11, 12]. The gold market and exchange rate of Iran had some movements affected by COVID-19 [13]. The economic growth was negative before the pandemic and the country faced the highest inflation rate in the past decade. This financial situation led to difficulties in financing prevention, diagnosis, and treatment services of COVID-19 [14, 15].

There are two primary methods for calculating CHE. The first method involves estimating general catastrophic health costs without specific disease attribution. This approach calculates CHE by assessing the total health expenditures relative to a measure of the ability to pay, such as total household spending. The Household Income and Expenditures Surveys (HIES) [7] is a good source for calculating the general catastrophic costs (CCC). Another method is calculating diseases-related CCC. In this study, we attempted to calculate COVID-19-related CCC. While national household surveys like the HIES track general health spending, they often overlook pandemic-specific costs such as income loss, preventive expenses, and informal coping strategies [16]. This implies that several estimations of the CHE using HIES data could potentially be lower than the actual figures [16]. To address these gaps, we conducted a dedicated COVID-19 survey designed to capture both direct and indirect household costs. Using regression modeling, we then extrapolated our findings to provinces not directly surveyed, providing a more comprehensive national estimate of COVID-19-related CCC that captures direct and indirect pandemic-related expenses not reflected in traditional datasets like HIES. Due to the lack of studies on the financial protection of COVID-19, our study provides a standard method for estimating the CCC. In addition, it suggested an approach for the prediction of the CCC in those regions in which there was a lack of COVID-19 expenditure surveys.

## 2. Methods

### 2.1. Study Design and Sampling Strategy

This was a population-based cross-sectional survey. This study applied a multistage random cluster sampling method. The districts were selected randomly for two district-level lists (urban and rural) of 10 Iranian provinces (Guilan, Tehran, Qazvin, Hormozgan, Khorasane-Razavi, Khuzestan, Fars, Kermanshah, Markazi, Ardebil). All of the selected 10 provinces represented one ethnic group or region of the country (Fars, Kurd, Arab, Jonubi, Tehrani, Gilak, and Turk). We randomly selected the sample blocks using the Iranian Statistical Center (ISC) GIS blocks. Each block contained between 600 and 800 households.

### 2.2. Participant Selection

We divided each block into 30-household clusters based on the location of the houses and conducted the interview for all household of each cluster. We arranged interviews for all households who agreed to participate in the study and accepted the informed consent. The selection process continued until there was a final sample for each block.

### 2.3. Survey Execution

**Figure 1:**
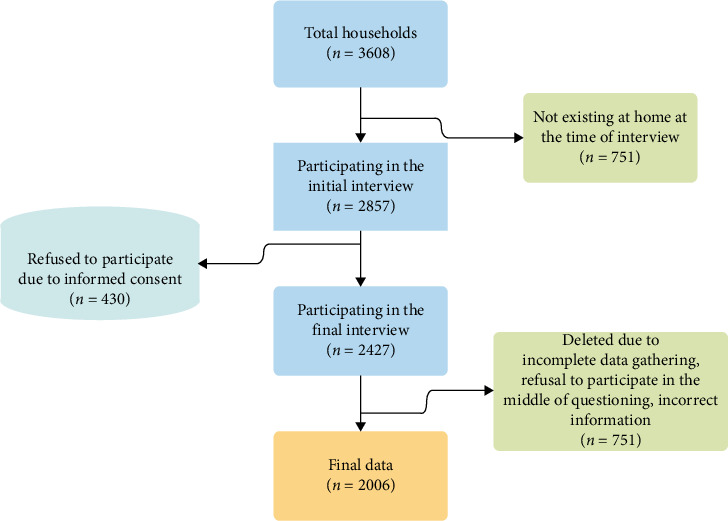
Sample selection flowchart: The flowchart shows we approached 3608 households, with 2006 completing the survey.

Figure 1 represents the data collection process. For the purpose of conducting interviews, 33 local interviewers were recruited and underwent training in four online courses. All households in a selected cluster were approached for participation. Households that provided informed consent were interviewed, and the sampling process continued until the target sample size for each block was achieved. In total, 3608 households were visited between May and December 2022, of which 2006 households (comprising 5844 individuals) completed the survey with valid and complete data. Interviews were conducted primarily with the household head or, in their absence, the person most informed about household income and expenditures. A total of 33 trained local interviewers conducted the fieldwork after completing four online training sessions.

### 2.4. Questionnaire Development

We initially developed the CCC questionnaire in Farsi/Persian, the official language. We assembled a multidisciplinary panel for semistructured interviews, including three health economists, three public health experts, two general physicians, five infectious disease specialists, two individuals who had COVID-19, three private sector employees, and five self-employed individuals. Through a series of semistructured interviews, panel members helped identify a wide range of cost elements associated with COVID-19, including direct medical and nonmedical expenses, income losses, and other out-of-pocket payments. These discussions were followed by an online focus group session to finalize the list of relevant cost categories and ensure that no key components were overlooked. Subsequently, we carried out a content validity assessment, including both the Content Validity Index/Ratio (CVI and CVR), for each cost item. This evaluation was performed in collaboration with 10 health experts specializing in health economics, infectious diseases, epidemiology, and health policy. Those items that did not reach a favorable score or those items which did not related to the study based on the scores of health experts were deleted from the questionnaire. In this phase, we asked the participants to add any additional local COVID treatment costs, which were likely, missed in the questionnaire. At the end, the final questionnaire was designed. The final questionnaire consisted of five sections:

*Introduction part*: This section included the inclusion and exclusion criteria, informed consent, and a treatment phase checklist (covering retreatment cases, new cases in the intensive treatment phase, and new cases in the continuation treatment phase).

*Overview of COVID-19 infection in the household (one year prior to the interview)*: It gathered data on the number of infected household members, the types of COVID-19 treatments received, and other related questions.

*COVID-19 treatment details (one year prior to the interview)*: This section covered information for both nonhospitalized and hospitalized patients, including hospitalization costs, drug expenses, job loss related to treatment, transportation costs, caregiver costs, caregiver job loss costs, and the use of herbal and complementary medicine related to COVID-19 treatment.

*Prevention costs (for one month prior to the interview)*: Participants reported expenses related to job loss due to COVID-19 lockdowns, purchasing masks, alcohol, and other preventive equipment, as well as costs of buying herbal products and fruits associated with COVID-19 prevention.

*Income and related variables*: This section included questions on changes in income after contracting COVID-19, the household's socioeconomic status and social position, health insurance coverage, self-reported household income, coping strategies, and household asset ownership.

Test-retest reliability showed a correlation of 78.8% between the two scores. We asked all the participants to show their health insurance card and invoices for the treatment of COVID-19 (if available). To ensure the quality of data, after ending the interviews for each interviewer, we selected phone numbers of interviewees randomly and asked them a part of the questionnaire. The accuracy of the gathered data was over 70%.

### 2.5. Statistical Analysis

The following formula was used to estimate the CCC faced by households:

(1) $$\begin{array}{c} T=\frac{\sum \text{OOPM}+\sum \text{OOPN}+\sum \text{IN}}{\sum y}, \end{array}$$

where ∑OOPM = monthly direct medical costs, ∑OOPN = monthly nonmedical costs, and ∑IN = monthly income loss related to COVID-19 and ∑*y* = prepandemic household monthly income. If *T* exceeded 20%, we defined household faced the CCC. We applied sampling weights (based on the total population of each province) to account for the multistage cluster sampling design and to ensure that the results were representative of the broader population across the 10 selected provinces.

We reanalyzed the results with and without considering the coping strategy. Finally, we employed a logistic regression model to determine the relationship between different variables and households facing CCC as follows:

(2) $$\begin{array}{c} {\text{CCC}}_{i}=\beta_{1}+\beta_{2}{\text{inp}}_{i}+\beta_{3}{\text{oup}}_{i}+\beta_{4}{\text{edu}}_{i}+\beta_{5}{\text{age}}_{i}+\beta_{6}{\text{men}}_{i}+\beta_{7}{\text{urb}}_{i}+\beta_{8}{\text{work}}_{i}+\beta_{9}{\text{bas}}_{i}+\beta_{10}{\text{comp}}_{i}+\beta_{11}{\text{wealth}}_{i}+\varepsilon_{i}, \end{array}$$

where CCC_*i*_ is the binary variable if households faced the CCC, inp_*i*_ is the binary variable of having one or more hospitalized persons due to COVID-19 in the household, oup_*i*_ is the binary variable of having one or more persons using outpatient services due to COVID-19 in the household, edu_*i*_ is the average years of education, men_*i*_ is the ordinal variable of the number of males in the household, urb_*i*_ is the binary variable of living in the urban or rural region, work_*i*_ is the ordinal variable of the number of people who have a paid work in the household, bas_*i*_ is the binary variable of having basic health insurance, comp_*i*_ is the binary variable of having complementary or supplementary health insurance, wealth_*i*_ is the wealth index calculated from household asset ownership. We calculated the wealth index for each household using principal component analysis (PCA) from the household living assets of 20 components (availability of television, vacuum machine, refrigerator, air conditioner, central heating system (three types of fan coil unit, central radiator heating system, package boiler system), home ownership type (three forms of rental, owner-occupied and employer-provided), home surface area, number of rooms, having car, bicycle, personal computer, washing machine, dishwasher, access to piped water, piped gas and internet). We had 61 missing data in the PCA analysis due to incomplete information of some components. The first principal component, which accounted for 15% of the total variance, was retained and used as the household wealth score.

We gathered data for 10 provinces. Using the value of the parameters in the regression model in equation (2), we predicted the CCC for the other provinces of the country. We utilized 2021 HIES for Iran conducted by the ISC in the estimation. We used STATA SE software V14.1 for data analysis. The National Institute for Medical Research Development of Iran has granted ethical approval for all study procedures (Ethics code: IR.NIMAD.REC.1400.121). The Iranian Rials (IRR) to US dollars exchange rate was 225,000 in this study (not adjusted monthly).

### 2.6. Sensitivity Analysis

The informal sector in Iran is predominant, so monthly self-reported household income might fall into bias. To avoid bias, we used a regression model to predict household annual income based on asset ownership and reanalyzed the results. For income prediction, we used 15 asset indicators including vehicles, electronics, and housing characteristics. Since CCC might be related to the prevalence of COVID-19, we re-estimated a province-level analysis to find changes in the prevalence of COVID-19 and the CCC. Furthermore, we tested thresholds from 10% to 50% to assess CHE sensitivity.

## 3. Results

### 3.1. Descriptive Statistics and Cost Burden

Table 1 shows the COVID-19-related health and medical costs in the households. The average monthly prevention costs of COVID-19 were 1.41 ± 2.0 US$. The average monthly inpatient and outpatient costs were 1.11 ± 4.87 and 3.65 ± 12.79 US$, respectively. The average monthly income loss costs were 0.06 ± 0.87 US$. The reason for low inpatient and outpatient cost was related to the inclusion of all samples (both those who were infected and those who were not) in the study. The average inpatient household costs was 10.95 ± 23.56 US$ for those households who had one or more inpatient and outpatient cases; the average inpatient costs were 138.74 US$ yearly if we consider households with hospitalized cases. The total household level cost of COVID-19 was 6.21 ± 13.92 US$ per month and, this figure was 17.14 ± 24.11 US$ for households who had one or more COVID-19 patients.

### 3.2. CCC Prevalence

Table 2 shows the percentage of people with the CCC. Of the 2006 households that participated in the study, 64 (3.19%) faced CCC if we add the coping strategies in the household income. Excluding the impact of coping strategies, the data reveals that 108 households (5.38%) are confronted with catastrophic financial costs.

Table 3 shows that the CCC varies between 1.86% for the Tehran Province and 6.9% for the Markazi Province. After removing coping strategies, the highest and lowest percentages of people facing the CCC were in Tehran (3.73%) and Markazi provinces (13.79%), respectively.

### 3.3. CCC Determinates

Table 4 shows the results of a logistic regression model to identify the relationship between individual and household level characteristics on the percentage of COVID-19-related CCC. The relationship between the wealth index and CCC is negative, (*β* = −0.187, 95% CI [−0.398 to 0.024]). Households with at least one member who received inpatient treatment for COVID-19 were more than tenfold as likely to face CCC (*β* = 2.324, 95% CI [1.65 to 2.997]), reflecting the high financial burden of hospitalization, outpatient service utilization also significantly increased the likelihood of CCC (*β* = 1.797, 95% CI [1.165 to 2.430]), suggesting even nonhospitalized COVID-19 cases contributed substantially to financial strain. The relationship between the average years of education of the household and CCC was negative and significant (*β* = −0.099, 95% CI [−0.192 to −0.005]), implying that the CCC was lower in households with a higher average education. Having basic health insurance (*β* = −0.678, 95% CI [−1.248 to −0.109]) or supplementary health insurance (*β* = −1.201, 95% CI [−2.612 to 0.210]) was associated with a lower risk of CCC, implying these mechanisms provided some degree of financial protection.

With the increase in the number of people over 65 years old in the household, the likelihood of the CCC increased significantly (*β* = 0.880, 95% CI [0.110 to 1.649]). Other variables did not have a significant relationship with CCC. We re-analyzed the results using the CCC without coping strategies. The relationship between the wealth index and the CCC was negative (*β* = −0.130, 95% CI [−0.294 to 0.034]). Inpatient (*β* = 1.926, 95% CI [1.367 to 2.485]) and outpatient (*β* = 1.511, 95% CI [1.009 to 2.013]) treatment was positive and significant, which indicates that households having one or more inpatient and outpatient treatment of COVID-19 had more CCC. The relationship between the average years of education of the family and the CCC was negative (*β* = −0.135, 95% CI [−0.210 to −0.061]) and significant. This means that in households with a higher average education had lower the CCC (without the existence of protective strategies). Additionally, the percentage of females in the household had a negative relationship with the CCC (without coping strategy), which was statistically significant (*β* = −0.736, 95% CI [−1.447 to −0.025]). The percentage of people over 65 years old in the household also had a significant coefficient (*β* = 0.720, 95% CI [0.106 to 1.334]). The CCC without protective strategies in people living in urban regions was more than that of rural residents (*β* = 0.534, 95% CI [0.063 to 1.006]). However, urban residence was not significantly associated with CHE when coping strategies were considered, suggesting that household-level financial responses may offset location-based differences under certain conditions. The CCC without coping strategies in households covered by insurance (*β* = −0.989, 95% CI [−1.432 to −0.547]) and covered people with supplementary insurance (*β* = −1.815, 95% CI [−3.212 to −0.419]) was less than others.

**Figure 2:**
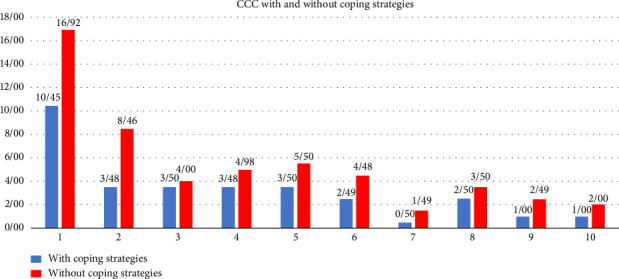
The CCC in different economic deciles with and without coping strategies.

Figure 2 shows the CCC in different economic deciles with and without coping strategies. With the first decile experiencing the highest burden 10.45% with coping strategies and 16.92% without coping strategies, while the seventh decile shows the lowest (0.5% with coping strategies and 1.49% without coping strategies), underscoring the disproportionate financial vulnerability of the poorest groups.

### 3.4. Sensitivity Analysis

**Figure 3:**
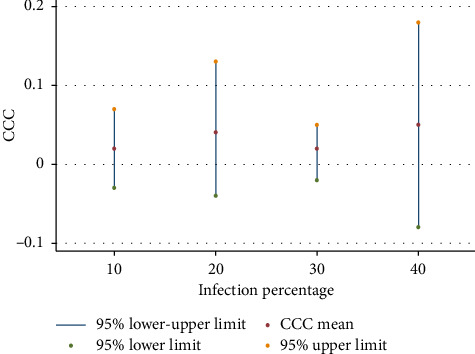
Changes in the CCC by different prevalence of COVID-19 in the provinces. Note: *Y*-axis shows the CCC and *X*-axis shows the infection percentages of 10%, 20%, 30%, and 40%.

We reanalyzed data by estimating the household income from the wealth index. The average estimated income was 270.6 US$ ±3.03. The average self-perceived income of household was 304.59 ± 5.48 US$ indicating that self-reported income may be understated, particularly among more vulnerable or informal-sector households. This aligns with prior evidence that self-reported income in low-income settings often underrepresents true earning capacity by considering the estimated income instead of the self-reported income of the households in the calculation of the CCC, we found that the CCC was 1.55%. In addition, we assessed changes in the CCC by changing the prevalence of COVID-19 in the provinces. As reported in Figure 3, results showed that in the 10%, 20%, 30%, and 40% prevalence of COVID-19, the CCC was 2%, 4%, 2%, and 5%, respectively, which showed no significant differences in different prevalence (*p* value of regression analysis in the relationship between the provincial level of infection and the CCC = 0.739) suggesting that utilization and cost, rather than infection rates alone, drive household financial burden.

**Figure 4:**
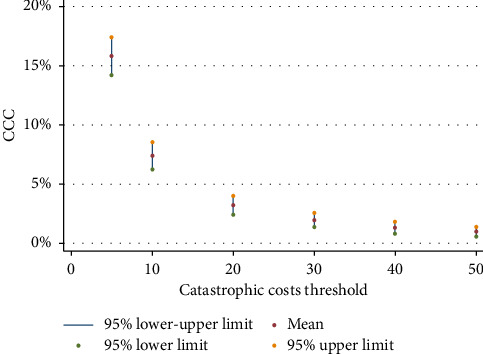
The CCC by varying level of the catastrophic cost's threshold. *Y*-axis shows the CCC and *X*-axis shows the catastrophic costs thresholds of 10%, 20%, 30%, 40%, and 50%.

The 20% threshold used in the study is the standard one which was recommended by the WHO [17]. However, to have comparable results, we used sensitivity analysis by changing the cost thresholds.  Figure 4 illustrates the varying levels of the CCC across different cost thresholds. When a 5% threshold is applied, the CCC stood at 15.8%. As the threshold increases to 10%, 20%, 30%, 40%, and 50%, the corresponding CHE rates were 7.4%, 3.2%, 1.9%, 1.2%, and 0.9%, respectively.  Figure 4's threshold analysis reveals that the estimated proportion of households facing COVID-19-related CCC drops sharply as the threshold increases highlighting how sensitive CCC estimates are to the selected cost definition.

### 3.5. Predicting the CCC in Other Provinces of Iran

**Figure 5:**
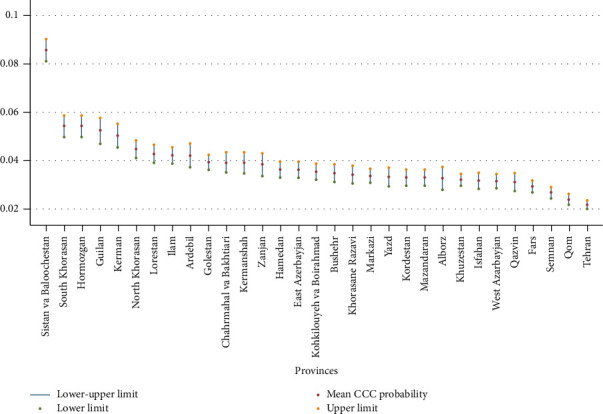
The estimated CCC in all provinces of Iran based on the HIES. Note: equation (2) was applied in the estimation.

Figure 5 displays the estimated CCC across Iranian provinces, calculated using the HIES data applied in equation (2). Sistan and Baluchistan province, located in the southeast of Iran, exhibited the highest CCC at 8.57%. This was followed by South Khorasan and Hormozgan, both at 5.4%, Guilan at 5.2%, Kerman at 5%, North Khorasan at 4.5%, and Lorestan at 4.2%. Conversely, the lowest CCC probabilities were observed in Tehran at 2.1%, Qom at 2.4%, Semnan at 2.6%, and Fars at 2.9%. The national average CCC was calculated to be 3.89%.

## 4. Discussion

### 4.1. Global Comparisons

This study focused on the COVID-19-related costs at the household level in Iran by measuring the CCC. The result suggested that the CCC in Iran was 3.89%. Significant proportion of Iranian households implemented coping strategies like selling their assets, getting loans to cope with the CCC. Besides the 3.89% of the CCC, approximately 2% of Iranian households would face CCC without implementing coping strategies. A previous study in Iran calculated the CCC in hospitalized patients and found that 61% of the cases faced the CCC in the delta variant of COVID-19 at a 40% threshold [18]. In a 2023 study in Indonesia, results showed that 18.6% of the sample faced CCC and 38.6% hospitalized people faced CCC at 40% threshold [19]. A time-series study across five countries assessed the CCC associated with COVID-19. It found CCC rates of 5% in Mexico, 13.5% in Belarus, 9.6% in Peru, 6.5% in Russia, and 11% in Vietnam. Although the study relied on national census data not specifically intended for COVID-19 cost analysis, the findings supported the hypothesis of a surge in CCC during the pandemic [20]. A South India study found the CCC rate of 49.7% at the 40% threshold [21]. Using household income and expenditures data, another study in Mexico calculated that 3% of households fall to CHE after COVID-19 [22]. The annual inpatient COVID-19 costs of participants were 138.74 US$. The inpatient cost in another study in Iran found to be 342.24 US$ in the current study exchange rate which contained medication, traditional treatment, homecare and Hemoperfusion [18]. However, in another study in Tehran, the mean cost of COVID-19 in hospitalized patients was 1434 US$ at 42,000 exchange rate (by considering 225,000 as the exchange rate the mean cost was 267.7 US$) [23]. The results showed a high level of heterogeneity in the CCC between countries. Differences in the methodologies for calculating the costs of COVID-19, especially in sample selection and defining the thresholds, can partially explain the heterogeneity in the CCCs; for example, many studies with high CCCs used hospital data in inclusion criteria. However, the type of health systems, the role of the private sector in the treatment of patients, and universal health coverage are other critical factors explaining heterogeneity in CCCs [24, 25]. The findings contained additional costs which were not imposed to the households before the COVID-19 outbreak. Facing the total CHE was varied between 6.2% and 9.1% before COVID-19 outbreak [26]. The results showed that coping strategies were an effective way to decrease CCC in Iran. A study in Egypt, Tunisia, Morocco, and Jordan showed that households sold their asset and borrowed from banks for their high costs of well-being during the COVID-19 pandemic [27].

### 4.2. Policy Implications

This study's findings indicate that CCC varied significantly across provinces, with the likelihood of incurring CCC being closely linked to a household's social class and the cost of services [28]. The substantial variation in CCC between reflects underlying disparities in healthcare infrastructure and financial protection mechanisms [29, 30]. Provinces like Markazi may have limited access to public health services, forcing households to rely on higher-cost private providers or informal markets. In contrast, Tehran benefits from more robust health infrastructure and wider insurance coverage, reducing the financial burden of COVID-19 care [31]. The Divergent social classes across provinces influenced the Catastrophic Cost Coefficients. Moreover, having access to basic and supplemental insurance played a pivotal role in CCC outcomes. These observations underscore the critical role of universal health coverage in providing financial protection for patients affected by COVID-19 [32]. In the estimated COVID-19 CCC analysis, Sistan and Baluchistan exhibited a particularly vulnerable condition. The estimated CCC was alarmingly high, with nearly 9% of the population facing CCC due to the pandemic. This situation is not surprising when considering the province's economic and social circumstances, which are characterized by high levels of household poverty and significant health and medical disadvantages [33–35]. In provinces like Sistan and Baluchistan, it is crucial for the government to implement social and health assistance programs. These should focus on providing essential health commodities to improve access to services and reduce both CCC and poverty. Achieving social justice and improving the health of community is one of the goals of social development [36]. All members of community should have access to the necessary and basic health and medical services. The best way to achieve these goals is financial protection against health issues [37]. The financial protection with universal health coverage is inevitable [38]. However, the health insurance system must be ready for emergency settings to prevent CCC in the health system shortages. For example, out-of-pocket payments for medication were extremely high, suggesting that patients resorted to purchasing drugs from the black market during COVID-19 outbreaks. In addition to formal protection mechanisms like insurance, households also relied on informal strategies such as borrowing and asset sales which is called coping strategies. Coping strategies are temporary solutions for households to protect them from facing CCC, but in the long term, they can affect the wealth, which might lead to household's financial hardship and poverty [39–41].

In an equitable health system, households participate in the financing of health services according to their financial and income abilities and do not pay more than their ability to pay [42]. Direct payments for health services might increase the risk of CCC [43]. The COVID-19 pandemic had important lessons for health policymakers about the efficacy of the health financing protection system in the crisis and shortage of health resources. The lack of hospital beds, and shortage of medications, vaccines, and medical equipment increased the direct payments of the patients for their health and well-being, empowered the private sector to raising the price of services, and increased CCC [24, 29]. To enhance financial protection during future health crises, three key policy measures are recommended. First, implement targeted insurance subsidies for low-income and informal-sector households, who face the highest risk of CCC. Second, enforce emergency price caps on essential COVID-19-related services and medications to prevent cost inflation during surges. Third, develop strategic stockpiles of critical medicines and protective equipment to ensure availability and stabilize prices, reducing the need for costly private or black-market purchases.

The findings of this study were in line with the emphasis that both inpatient and outpatient payments for COVID-19 increased the likelihood of the CCC. The CCC was higher in low-income groups which indicated that access to the protective health services and health insurances was limited in these groups. The main aim of insurance systems is to redistribute the financial resource from the rich to the poor [44]. The relatively low CCC suggests that, overall; people did not experience severe financial strain due to COVID-19. Nonetheless, a notable CCC difference persisted between the country's poorer and wealthier populations. The government tried to do some interventions to control the CCC for the poor, like free health insurance for those who did not have access to health insurance [45, 46]. Furthermore, health insurance coverage is not adequate when there is a gap between supply and demand of medications and medical services [47–51]. In private market cases, the price of services might become too high that patients cannot pay for the services [24, 29, 52]. The regression results confirmed that the role of complementary health insurance is important for financial protection of such cases. For public sector health services, there are regulations which do not allow the market to increase the prices. Thus, supply and demand gap might lead to the lack of services, especially in low- and middle-income countries [30, 31, 53]. In such cases, despite the ability to pay for services, no services are available for utilization. The policymakers suggest public health implementations to decrease or delay the demand for services. Some other implementations, like reducing the quality of services and coverage of essential and basic health service packages, are effectively practical in increasing the supply of services [49, 54, 55].

In addition to the role of insurance and out-of-pocket payments, structural characteristics of Iran's health system significantly influenced the extent of CCC during the COVID-19 pandemic. Access to affordable public healthcare services—particularly medications, diagnostic tools, and hospital beds—were constrained in many regions in the COVID-19 outbreak. The shortage of hospital beds and intensive care capacity forced some patients to seek care in private facilities, where costs were considerably higher and often not fully covered by insurance schemes [31, 51]. This shift toward the private sector, in the context of emergency demand surges, contributed directly to increased out-of-pocket expenditures, particularly for vulnerable households.

Moreover, essential medications were sometimes unavailable through public channels, prompting patients to turn to informal or black markets where prices were inflated and supply was unreliable [29]. These dynamics disproportionately affected low-income households, who were less able to absorb high costs or access private-sector care. Even among insured households, financial protection was limited when services or medications were unavailable, revealing a key vulnerability in health system preparedness [14, 24]. These findings highlight that financial protection alone is not enough; the availability and accessibility of quality public health services are essential to prevent CCC during health crises [55].

The results showed that more educated groups faced less CCC. A systematic review analyzed the relationship between educational status and facing CHE and found that in most studies, low education had a relationship with a higher probability of CCC [56]. In addition, being senior increased the risk of CCC. This finding is confirmed in the literature in the non-COVID-19 CCC studies [56, 57].

Iran's relatively low CCC estimate of 3.89%, may be partly explained by government interventions, such as emergency price controls, free COVID-19 services in public hospitals, and the expansion of basic insurance coverage during the pandemic. These measures likely helped cushion households from the full financial impact of care, even amid widespread inflation and supply constraints. Although this study focused on household-level determinants of CCC, broader systemic factors likely played a substantial role. Government emergency responses, such as the scale and speed of testing, treatment, and vaccination, could have either reduced or intensified financial burdens [51, 55]. Limited healthcare infrastructure and poor coordination between national and provincial authorities often pushed patients toward costlier private or informal care [31]. Additionally, lockdowns and economic disruptions disproportionately affected informal workers, increasing vulnerability [58]. These external factors, though not directly captured in the study, likely shaped the variation in CCC and should be considered in future research.

### 4.3. Limitations

A significant limitation of the study stemmed from the timing of data collection, which coincided with a period of low COVID-19 prevalence that varied widely among provinces. However, our analysis showed that the CCC was not sensitive to the prevalence of COVID-19 infection. A limitation of this study pertains to the reliance on aggregated household-level data, which might constrain the analysis. We encountered challenges in collecting detailed household cost and income data at both the individual and provincial levels. Consequently, significant factors that may have influenced variations in the CCC across provinces during the pandemic (such as access to information, social dynamics, religious affiliations, and political preferences) were excluded from the analysis. Another limitation was related to the CCC threshold. Several studies used 40% of household income as the catastrophic threshold. However, the WHO suggested 20% as the threshold for another infective disease as a standard threshold [17]. To have comparable results, we used another sensitivity analysis by using different levels of CCC threshold. Calculating the costs of long COVID-19 is another limitation of this study which needs further research. At the time of study, the side effects of COVID-19 and long COVID disorders were not specified completely. The researchers and respondents were not able to distinguish between the long COVID-19 and other diseases. Thus, we eliminate adding long COVID-19 from the results. Furthermore, the CCC is highly related to the utilization of services. Many household might not face CCC because of not utilizing health services and not having out-of-pocket payments. They might need the health services, but because of their inability to pay for services, they did not use health services. This condition is not rare, especially in low-income groups.

## 5. Conclusion

The COVID-19 pandemic resulted in increased financial burdens on households. In response, health financing protection systems should offer specialized financing packages tailored for emergencies like COVID-19. However, it is crucial to note that these packages might not yield effective outcomes if they fail to balance supply and demand during outbreak situations.

## Figures and Tables

**Table 1:** Prevention, outpatient and inpatient costs, and income loss for the total population (*n* = 2006) and for those households who had COVID-19 inpatient or outpatient visits (*n* = 464).

Variable	Observations	Mean^∗^	Standard error	Minimum	Maximum
*Total population*					
Prevention costs	2006	1.413	2.009	0	20.889
Outpatient costs	2006	3.649	12.791	0	360.000
Inpatient costs	2006	1.108	4.879	0	57.778
Income loss costs	2006	0.059	0.870	0	25.778

*Households who had inpatient or outpatient visits only*					
Prevention costs	464	1.332	2.011	0	20.889
Outpatient costs	464	10.952	23.556	0	360.000
Inpatient costs	464	4.790	9.242	0	57.778
Income loss costs	464	0.143	1.190	0	24.000

^∗^Values are reported in US$.

**Table 2:** Percentage of the CCC with and without coping strategies.

Variable	Frequency	Percentage	Frequency	Percentage
	With coping strategies		Without coping strategies	
Not facing CCC^∗^	1942	96.81	1898	94.62
Facing CCC	64	3.19	108	5.38

*Note:* Estimations are based on equation (1).

^∗^The CCC: COVID-19 related catastrophic costs.

**Table 3:** The percentage of households faced by the CCC in the selected provinces with and without coping strategies.

Variable	Ardebil	Fars	Guilan	Khorasan Razavi	Khoozestan	Markazi	Hormozgan	Kermanshah	Qazvin	Tehran	Total sample
*With coping strategies*											
Not facing CCC	124	147	330	106	114	54	129	50	98	790	1942
Facing CCC	3	6	17	4	4	4	7	2	2	15	64
CCC percentage	2.36	3.92	4.90	3.64	3.39	6.90	5.15	3.85	2.00	1.86	3.19

*Without coping strategies*											
Not facing CCC	119	144	321	105	112	50	128	49	95	775	1898
Facing CCC	8	9	26	5	6	8	8	3	5	30	108
CCC percentage	6.30	5.88	7.49	4.55	5.08	13.79	5.88	5.77	5.00	3.73	5.38

**Table 4:** The results of a logistic regression model to identify the relationship between individual and household level characteristics on the percentage of CCC with and without coping strategies.

Variables^∗∗∗^	The CCC			The CCC without coping strategies		
	Coefficient (*β*)	Standard error	95% lower-upper limit	Coefficient (*β*)	Standard error	95% lower-upper limit
wealth_*i*_	−0.187^∗^	0.108	−0.398 to 0.024	−0.130^∗^	0.084	−0.294 to 0.034
inp_*i*_	2.324^∗∗^	0.344	1.650 to 2.997	1.926^∗∗^	0.285	1.367 to 2.485
oup_*i*_	1.797^∗∗^	0.323	1.165 to 2.430	1.511^∗∗^	0.256	1.009 to 2.013
edu_*i*_	−0.099^∗∗^	0.048	−0.192 to −0.005	−0.135^∗∗^	0.038	−0.210 to −0.061
age_*i*_	0.880^∗∗^	0.393	0.110 to 1.649	0.720^∗∗^	0.313	0.106 to 1.334
men_*i*_	−0.420	0.446	−1.294 to 0.454	−0.736^∗∗^	0.363	−1.447 to −0.025
urb_*i*_	0.433	0.301	−0.158 to 1.024	0.534^∗∗^	0.240	0.063 to 1.006
work_*i*_	−0.337	0.387	−1.096 to 0.422	−0.379	0.309	−0.985 to 0.227
bas_*i*_	−0.678^∗∗^	0.291	−1.248 to −0.109	−0.989^∗∗^	0.226	−1.432 to −0.547
comp_*i*_	−1.201^∗^	0.720	−2.612 to 0.210	−1.815^∗∗^	0.713	−3.212 to −0.419
constant	−3.283^∗∗^	0.553	−4.367 to −2.198	−1.852^∗∗^	0.413	−2.661 to −1.044

*Note:* Estimations are based on equation (1).

^∗^Significant at 90% confidence interval.

^∗∗^Significant at 95% confidence interval.

^∗∗∗^Variable abbreviations are discussed in equation (2) in methods section.

## Data Availability

The data that support the findings of this study are available on request from the corresponding author. The data are not publicly available due to privacy or ethical restrictions.
