# Corrigendum to “The COVID-19 Pandemic Economic Implications in Iran: A National Survey Assessing Catastrophic Health Expenditures”

**DOI:** 10.1155/ipid/9862748

**Published:** 2025-09-19

E. Homaie Rad, M. Hajizadeh, S. Yousefzadeh-Chabok, et al., “The COVID-19 Pandemic Economic Implications in Iran: A National Survey Assessing Catastrophic Health Expenditures,” *Interdisciplinary Perspectives on Infectious Diseases* 2025 (2025): 8854646, https://doi.org/10.1155/ipid/8854646.

In the article titled “The COVID-19 Pandemic Economic Implications in Iran: A National Survey Assessing Catastrophic Health Expenditures,” there was an error in affiliation 17. The correct affiliation is shown below:

Health Human Resources Research Center, School of Health Management and Information Sciences, Shiraz University of Medical Sciences, Shiraz, Iran.

We apologize for this error.

